# Intermittently scanned CGM in older adults with type 2 diabetes not taking insulin: iMMEDIATE post hoc analysis

**DOI:** 10.1210/jendso/bvag004

**Published:** 2026-01-13

**Authors:** Caity Decara, Annis Marney, Samiollah Gholam, Joan Skelly, Matthew P Gilbert

**Affiliations:** Robert Larner, M.D. College of Medicine, University of Vermont, Burlington, VT 05401, USA; Frist Clinic, Nashville, TN 37203, USA; Division of Endocrinology and Diabetes, Robert Larner, M.D. College of Medicine, University of Vermont, Burlington, VT 05401, USA; Department of Biomedical Statistics, University of Vermont, Burlington, VT 05401, USA; Division of Endocrinology and Diabetes, Robert Larner, M.D. College of Medicine, University of Vermont, Burlington, VT 05401, USA

**Keywords:** continuous glucose monitoring, type 2 diabetes, older adults, time in range

## Abstract

**Introduction:**

In a post hoc analysis of data from the IMMEDIATE study, we compared mean percentage time in range (TIR) in study participants with T2D aged <65 and ≥65 years who were randomized to intermittent subcutaneous continuous glucose monitoring (isCGM) plus diabetes self-management education (DSME) vs DSME alone.

**Methods:**

Glucose metrics were analyzed using a linear mixed-effects model, with fixed effects for treatment and age group and random effect for study site. Baseline HbA1c was included as a covariate. Patient-reported outcomes were also evaluated.

**Results:**

The majority of trial participants were aged <65 years, but percentages of adults aged <65 and ≥65 years were similar in the isCGM + DSME and DSME groups. When compared between age groups, mean differences in glycemic outcomes were not statistically significant. Hypoglycemia was infrequent. Among patients aged <65 years, a mean difference of 0.57 (95% CI, 0.38-0.77) in the Glucose Measurement Satisfaction Survey score was observed in participants receiving isCGM + DSME vs DSME; the difference was 0.12 (95% CI, −0.23 to 0.48) in adults aged ≥65 years, with a significant treatment-by-age group interaction (*P* = .03). No significant treatment-by-age group interactions were observed for the Diabetes Distress Scale or other patient-reported outcomes.

**Conclusion:**

Our findings support the use of isCGM plus DSME to increase TIR in adults with T2D who are not using insulin, independent of age.

In the United States, nearly 1 in 3 adults aged 65 years and older have diabetes (29.2%), while almost half of older adults (48.8%) are diagnosed with prediabetes [1]. As the ageing population in the U.S. continues to grow, the prevalence of diabetes and prediabetes presents a substantial public health challenge.

Management of type 2 diabetes (T2D) often requires multiple medications, including combinations of oral and injectable formulations. Polypharmacy in older adults significantly increases the risk of adverse events, including hypoglycemia and medication interactions, which are particularly concerning in an ageing population [2-4].

Despite having an increased prevalence of diabetes, older adults are frequently excluded from clinical trials, perpetuating a critical gap in knowledge of the safety and efficacy of new and/or established treatments in this age group [3]. Regulatory bodies have recommended collecting comprehensive data [5, 6].

To address these management challenges, continuous glucose monitoring (CGM) offers several benefits for patients with T2D, such as real-time glucose tracking, improved glucose control, and alerts for high and low glucose levels. Continuous glucose monitoring significantly alleviates the burden of traditional fingerstick testing, thus reducing inconvenience. Previous studies have shown that the use of CGM improves glycemic outcomes, with decreased mean HbA1c levels and decreased glycemic variability in older adults with T2D who use multiple daily injections of insulin [7, 8]. A recent trial demonstrated how CGM is also effective for adults with T2D using basal insulin and other non-insulin medications [9]. Although CGM initiation has increased over time, it is used by <10% of insulin-treated older adults with T2D and those aged >85 years are even less likely to receive a CGM [10]. Data are also limited regarding the efficacy of CGM for older adults with T2D who are not treated with insulin. In addition, insurance programs, such as the Centers for Medicare and Medicaid Services (CMS), only cover CGM for patients with T2D using insulin, thus making evaluation of the non-insulin using CGM population more difficult.

The IMpact of flash glucose Monitoring in pEople with type 2 Diabetes Inadequately controlled with non-insulin Antihyperglycaemic ThErapy (IMMEDIATE) study (NCT04562714) explored the effect of intermittent subcutaneous continuous glucose monitoring (isCGM) combined with diabetes self-management education (DSME) vs DSME alone in patients treated with non-insulin medications for the management of T2D. The study demonstrated significant improvements in glycemic control. At 16 weeks of follow-up, time in range (TIR) in the isCGM + DSME group was 9.9% longer than TIR in the DSME group, while time above range (TAR) was shorter, and the reduction in HbA1c was greater compared with DSME alone [11]. Given the potential influence of age on adoption of CGM, we conducted a post hoc analysis of the IMMEDIATE trial data to further assess efficacy and safety data in older adults by comparing the differences in mean percentage TIR for participants <65 and ≥65 years of age. We also examined patient-reported outcomes (PROs) in an effort to evaluate age-related differences in health-related quality of life associated with CGM.

## Methods

### Study design

The IMMEDIATE study (NCT04562714) was a randomized, open-label study comparing isCGM plus DSME with DSME alone in 116 adults with T2D and an HbA1c ≥ 7.5% who were treated with non-insulin agents (1:1 randomization). Over a 16-week randomization period, both study groups received the same structured DSME and were instructed to conduct fingerstick self-monitoring of blood glucose (SMBG) at least 4 times daily. The primary outcome of the main study was the difference in percentage TIR between treatment groups, and key secondary outcomes included between-group differences in mean HbA1c, time below range (TBR), and TAR [11]. PROs were measured with the Glucose Measurement Satisfaction Survey (GMSS; score range 0-5, with a higher score indicating greater glucose monitoring satisfaction); Diabetes Distress Scale (DDS; score range 0-6, with a higher score indicating greater distress); Adherence to Refills and Medications–Diabetes (ARMS-D; score range 11-44, with a higher score indicating better adherence); and the Skills, Confidence and Preparedness Index (SCPI; score range 0-7, with a higher score indicating better self-management) [11-14]. IMMEDIATE was conducted in accordance with the Declaration of Helsinki and with the ethical principles of the International Council on Harmonization Good Clinical Practice Guidelines. An independent ethics committee approved the study protocol, and all participants provided written informed consent. The design and results from the main study have been previously published [11].

### Study population

The data set used in the present analysis included 108 patients for whom HbA1c data were collected (54 patients in each treatment arm). Participants with CGM data with <70% of wear time were excluded from the analysis of CGM endpoints (isCGM + DSME, n = 51; DSME-only, n = 48).

### Outcomes

The present post hoc analysis compared findings in subgroups of patients aged <65 and ≥65 years who were assigned to isCGM + DSME or DSME alone. Outcomes reported here include TIR (3.9-10.0 mmol/L), time in tight glycemic range (ie, 3.9-7.8 mmol/L), TAR (>10.0 mmol/L), TBR (<3.9 mmol/L), TBR level 2 (<3.0 mmol/L), mean blood glucose, coefficient of variation (CV), hypoglycemic events (<3.9 mmol/L and <3.0 mmol/L), GMSS, DDS, ARMS-D, and SCPI.

### Statistical analysis

The isCGM metrics and PRO measures at follow-up were analyzed using a linear mixed-effects model, with fixed effects for treatment, age group, and the interaction of treatment and age group; random effect for study site; and baseline HbA1c as a covariate. For the PRO measures, the models also included baseline total score as a covariate. If the interaction was not significant, it was dropped from the model. For all outcomes, the change from baseline to follow-up was calculated by subtracting the follow-up value from the baseline values. All analyses were run using SAS v9.4 (SAS Institute Inc., Cary, NC).

## Results

Table 1 displays the baseline characteristics and medications used for 108 participants in the IMMEDIATE trial divided into subgroups of patients <65 and ≥65 years of age. More study participants were aged <65 (n = 82) than ≥65 years (n = 26), and there were more men (n = 68) than women (n = 40). However, the percentage of men and women and those <65 and ≥65 was similar in the isCGM + DSME and DSME-alone groups. Patients aged ≥65 years had a lower baseline A1C in both arms of the study. There were no differences in the percentage use of concomitant medications in patients <65 or ≥65 years in either arm of the study.

**Table 1:** Baseline characteristics of the isCGM + DSME and DSM-only cohorts stratified by age

Characteristic mean ± SD or n (%)	isCGM + DSME		DSME	
	<65 years (n = 40)	≥65 years (n = 14)	<65 years (n = 42)	≥65 years (n = 12)
Age (years)	55.0 ± 8.0	70.6 ± 3.8	53.8 ± 9.2	70.0 ± 5.5
Duration of T2D (years)	8.0 ± 5.7	13.7 ± 6.7	11 ± 6.1	11.2 ± 4.7
Female	15 (37.5)	5 (35.7)	16 (38.10)	4 (33.3)
HbA1c (%)	8.6 ± 1.1	8.1 ± 0.5	8.7 ± 1.3	8.2 ± 0.7
% TIR (3.9-10.0 mmol/L)	53.3 ± 28.5	63.8 ± 21.4	57.5 ± 26.2*^a^*	63.7 ± 23.6
% TAR (>10.0 mmol/L)	45.9 ± 29.4	33.7 ± 23	40.3 ± 27.8*^a^*	33.0 ± 25.1
% TBR (<3.9 mmol/L)	0.7 ± 1.3	2.3 ± 4.9	1.3 ± 3.1*^a^*	2.3 ± 4.3
Glucose (mmol/L)	10.4 ± 3.1	9.1 ± 1.9	9.8 ± 2.8*^a^*	8.9 ± 2.2
Weight (kg)	88.4 ± 19.1	78.5 ± 15.9	85.9 ± 23.9	80.8 ± 18.4
Body mass index (kg/m^2^)	30.8 ± 6.7	28.3 ± 3.8	29.8 ± 6.7	28.8 ± 4.1
Waist circumference (cm)	103.9 ± 15.3*^c^*	101.6 ± 10.9	101.7 ± 18*^b^*	97.9 ± 12.9
Systolic blood pressure (mmHg)	127.1 ± 13.0	127.9 ± 14.9	124.3 ± 15.2*^a^*	128.4 ± 18
Diastolic blood pressure (mmHg)	83.1 ± 9.9	75.9 ± 9.3	81.9 ± 12	79.4 ± 11.6
eGFR (mL/min/1.73 m^2^)	92.3 ± 20.3	78.2 ± 14.2	94.1 ± 14.8	72.9 ± 15.6
Non-insulin antihyperglycemic therapies	2.5 ± 0.7	2.8 ± 1	2.7 ± 1	3.0 ± 1.0
Metformin	40 (100.0)	14 (100.0)	40 (95.2)	12 (100.0)
Sulfonylurea	21 (52.5)	9 (64.3)	19 (45.2)	5 (41.7)
SGLT2i	14 (35.0)	5 (35.7)	18 (42.9)	5 (41.7)
DPP-4i	15 (37.5)	6 (42.9)	20 (47.6)	7 (58.3)
GLP-1 RA	11 (27.5)	5 (35.7)	15 (35.7)	4 (33.3)

Abbreviations: DPP-4i, dipeptidyl peptidase 4 inhibitor; DSME, diabetes self-management education; eGFR, estimated glomerular filtration rate; GLP-1 RA, glucagon-like peptide 1 receptor agonist; isCGM, intermittently scanned continuous glucose monitoring; SD, standard deviation; SGLT2i, sodium-glucose cotransporter 2 inhibitor; TAR, time above range; TBR, time below range; TIR, time in range; T2D, type 2 diabetes.

^*a*^n = 41.

^*b*^n = 40.

^*c*^n = 38.

The interaction of treatment and age group was not significant for any of the glycemic outcomes. As shown in Table 2, the results for the main effect of treatment for each of the glycemic outcomes were consistent with those reported in the main study. When compared between age groups, mean differences in glycemic outcomes were not statistically significant. Hypoglycemia was infrequent. In the isCGM + DSME arm, the median number of hypoglycemic events was 1 each in the <65 and ≥65 year subgroups, whereas in the DSME-only arm, the median was 0 events in those aged <65 years and 1.5 in the ≥65 year subgroup. The median number of level 2 hypoglycemic events was 0 for all age and treatment groups.

**Table 2:** End of study CGM metrics between the isCGM + DSME and the DSME alone arms stratified by age

isCGM metric	isCGM + DSME		DSME		DSME vs isCGM + DSME		<65 vs ≥65 years	
	<65 years (n = 40)	≥65 years (n = 11)	<65 years (n = 36)	≥65 years (n = 12)	Adjusted mean difference (95% CI)	*P* value	Adjusted mean difference (95% CI)	*P* value
% TIR (3.9-10.0 mmol/L)								
Baseline, mean ± SD	53.3 ± 28.5	67.7 ± 18.9	58.4 ± 25.2	63.7 ± 23.6				
Change from baseline, mean ± SD	20.8 ± 29.0	16.6 ± 16.9	4.3 ± 20.7	9.5 ± 30.8				
End of study, mean ± SD	74.1 ± 17.9	84.3 ± 13.1	63.0 ± 23.9	73.3 ± 16.4	10.0 (2.7 to 17.4)	.01	−6.5 (−15.2 to 2.2)	.14
% time in tight glycemic range (3.9-7.8 mmol/L)								
Baseline, mean ± SD	30.7 ± 25.4	38.2 ± 20.1	32.8 ± 23.1	36.6 ± 23.7				
Change from baseline, mean ± SD	16.7 ± 26.6	22.7 ± 26.8	5.4 ± 19.9	10.2 ± 32.0				
End of study, mean ± SD	47.4 ± 22.2	60.9 ± 18.0	38.3 ± 23.6	46.8 ± 21.3	8.8 (0.6 to 16.9)	.03	−6.6 (−16.4 to 3.2)	.18
% TAR (>10.0 mmol/L)								
Baseline, mean ± SD	45.9 ± 29.4	29.1 ± 20.5	39.0 ± 27.0	33.0 ± 25.1				
Change from baseline, mean ± SD	−22.7 ± 27.9	−15.0 ± 19.0	−5.4 ± 21.2	−9.7 ± 32.0				
End of study, mean ± SD	23.2 ± 19.0	14.1 ± 12.6	33.2 ± 26.3	23.3 ± 17.1	−8.3 (−15.9 to −0.7)	.03	5.0 (−4.1 to 14.2)	.28
% TBR (<3.9 mmol/L)								
Baseline, mean ± SD	0.7 ± 1.3	2.8 ± 5.5	1.5 ± 3.3	2.3 ± 4.3				
Change from baseline, mean ± SD	1.3 ± 3.3	−1.4 ± 4.1	1.7 ± 6.1	0.06 ± 1.3				
End of study, mean ± SD	2.0 ± 3.8	1.4 ± 2.2	3.2 ± 7.1	2.4 ± 4.1	−1.3 (−3.3 to 0.8)	.22	1.1 (−1.3 to 3.5)	.37
% TBR level 2 (<3.0 mmol/L)								
Baseline, mean ± SD	0.2 ± 0.5	0.4 ± 1.1	1.1 ± 4.4	1.0 ± 2.1				
Change from baseline, mean ± SD	0.6 ± 2.6	−0.3 ± 1.2	−0.3 ± 1.2	0.08 ± 1.6				
End of study, mean ± SD	0.8 ± 2.6	0.1 ± 0.2	0.8 ± 3.3	1.1 ± 2.3	−0.3 (−1.4 to 0.7)	.54	0.4 (−0.9 to 1.7)	.50
Glucose (mmol/L)								
Baseline, mean ± SD	10.4 ± 3.1	8.7 ± 1.7	9.7 ± 2.8	8.9 ± 2.2				
Change from baseline, mean ± SD	−2.1 ± 2.9	−1.1 ± 1.6	−0.6 ± 2.5	−0.7 ± 2.6				
End of study, mean ± SD	8.2 ± 1.7	7.6 ± 1.0	9.0 ± 2.7	8.2 ± 1.6	−0.6 (−1.3 to 0.1)	.11	0.3 (−0.6 to 1.2)	.45
CGM SD (mmol/L)								
Baseline, mean ± SD	2.7 ± 0.8	2.4 ± 0.5	2.6 ± 0.7	2.3 ± 0.6				
Change from baseline, mean ± SD	−0.5 ± 0.5	−0.4 ± 0.4	−0.2 ± 0.6	−0.01 ± 0.4				
End of study, mean ± SD	2.2 ± 0.6	2.0 ± 0.6	2.4 ± 0.6	2.3 ± 0.7	−0.2 (−0.4 to 0.0)	.11	0.1 (−0.2 to 0.4)	.51
CV (%)								
Baseline, mean ± SD	27.2 ± 6.8	28.0 ± 8.2	27.9 ± 7.1	27.6 ± 9.6				
Change from baseline, mean ± SD	0.4 ± 4.4	−1.6 ± 4.0	−0.2 ± 5.0	1.2 ± 3.8				
End of study, mean ± SD	27.6 ± 7.0	26.4 ± 6.6	27.9 ± 6.9	28.7 ± 8.0	−0.7 (−3.5 to 2.2)	.65	0.7 (−2.6 to 4.1)	.67
No. hypoglycemic events, median (IQR)	1 (0.0, 5.5)	1 (0.0, 5.0)	0 (0.0, 5.0)	1.5 (0.0, 6.5)				
No. level 2 hypoglycemic events, median (IQR)	0 (0.0, 1.0)*^a^*	0 (0.0, 1.0)	0 (0.0, 1.0)	0 (0.0, 2.0)				
No. nocturnal hypoglycemic events, median (IQR)	0 (0.0, 2.5)	0 (0.0, 3.0)	0 (0.0, 4.5)	0 (0.0, 2.5)				

Abbreviations: CI, confidence interval; CV, coefficient of variation; DSME, diabetes self-management education; IQR, interquartile range; isCGM, intermittently scanned continuous glucose monitoring; SD, standard deviation; TAR, time above range; TBR, time below range; TIR, time in range.

^*a*^n = 39.

Glucose Measurement Satisfaction Survey was the only PRO outcome with a significant treatment-by-age group interaction (*P* = .03). Among patients <65 years, mean GMSS score was significantly higher in the isCGM + DSME arm compared with the DSME arm, with a mean difference of 0.57 (95% CI, 0.38-0.77). Among patients aged ≥65 years, the mean difference of 0.12 was not significant (95% CI, −0.23 to 0.48). Consistent with the main study, treatment group differences were not statistically significant for the other PROs. PRO findings were not significantly different between age subgroups (Table 3).

**Table 3:** PROs in the isCGM + DSME and DSME alone arms stratified by age

	<65 years		≥65 years		*P* values		
	isCGM + DSME (n = 38)	DSME (n = 40)	isCGM + DSME (n = 12)	DSME (n = 11)	Treatment arm	Age group	Treatment arm × age group
GMSS, mean ± SD							
Baseline	3.42 ± 0.50	3.26 ± 0.54	3.39 ± 0.59)	3.57 ± 0.37			
Change from baseline	0.56 ± 0.57	0.08 ± 0.54	0.38 ± 0.39	0.15 ± 0.33			
End of study	3.98 ± 0.43	3.34 ± 0.50	3.77 ± 0.60	3.72 ± 0.34	<.001	.89	.03
Adjusted mean difference (95% CI)	0.57 (0.38 to 0.77)		0.12 (−0.23 to 0.48)				
DDS, mean ± SD							
Baseline	2.46 ± 1.15	2.45 ± 1.11	1.86 ± 0.65	1.72 ± 0.68			
Change from baseline	−0.35 ± 1.19	−0.43 ± 1.31	−0.24 ± 0.77	−0.20 ± 0.75			
End of study	2.12 ± 1.17	2.02 ± 1.06	1.62 ± 0.63	1.52 ± 0.53	.38	.63	.98
Adjusted mean difference (95% CI)	0.17 (−0.25 to 0.59)		0.16 (−0.60 to 0.91)				
ARMS-D, mean ± SD							
Baseline	14.13 ± 2.74	16.10 ± 3.84	12.58 ± 1.44	13.82 ± 1.66			
Change from baseline	−0.29 ± 2.40	−0.23 ± 4.10	0.75 ± 2.14	−0.27 ± 2.45			
End of study	13.84 ± 2.79	15.88 ± 4.95	13.33 ± 2.93	13.55 ± 2.62	.60	.83	.38
Adjusted mean difference (95% CI)	−0.63 (−2.07 to 0.81)		0.65 (−1.91 to 3.20)				
SCPI, mean ± SD							
Baseline	5.35 ± 0.84	5.25 ± 0.91	5.39 ± 0.69	5.34 ± 1.24			
Change from baseline	0.82 ± 0.84	0.77 ± 0.78	0.65 ± 0.62	0.86 ± 0.76			
End of study	6.18 ± 0.74	6.02 ± 0.81	6.03 ± 0.80	6.20 ± 1.03	.89	.63	.35
Adjusted mean difference (95% CI)	0.08 (−0.21 to 0.38)		−0.20 (−0.74 to 0.34)				

Abbreviations: ARMS-D, Adherence to Refills and Medications–Diabetes; DDS, Diabetes Distress Scale; DSME, diabetes self-management education; GMSS, Glucose Measurement Satisfaction Survey; isCGM, intermittent subcutaneous continuous glucose monitoring; PRO, patient-reported outcome; SCPI, Skills, Confidence and Preparedness Index.

## Discussion

Investigation into the efficacy and tolerability of interventions for older adults is essential given the additional challenges older adults with diabetes encounter, including polypharmacy, cost and availability of systems, and unfamiliarity with new technologies [15]. Addressing these concerns is critical to ensuring that older adults have access to valuable diabetes management tools that can improve their quality of life. Integrating technologies such as CGM, insulin pumps, Bluetooth-enabled insulin pens, and closed-loop systems (CGM + insulin pumps) has the potential to provide a higher quality of diabetes care and empower patients by minimizing the burden of blood glucose monitoring with fingersticks and enabling a timely response to blood glucose changes. At the forefront of many researchers’ minds in the field of geriatric diabetes is the question, how does the effectiveness of diabetes technology vary across subgroups of older adults (≥65 years of age), given possible differences in overall health status, number of diabetes-related complications, and potential dependence on others due to physical or cognitive limitations [16]. There is also interest in clinicians’ understanding of patients’ beliefs and attitudes about monitoring tools. The GMSS is a validated questionnaire used to assess patient satisfaction with glucose monitoring systems, evaluating various dimensions including openness, emotional burden, behavioral burden, and trust [17]. Notably, older adults with cognitive or functional impairments have been under-represented in clinical trials of CGM, highlighting a critical need for more inclusive research in this population. However, the IMMEDIATE trial included adults ≥65 years of age, offering a valuable opportunity to explore outcomes in this population, prompting this post hoc analysis.

At 16 weeks of follow-up in the IMMEDIATE trial, the isCGM + DSME arm had significantly better outcomes compared to DSME alone. TIR was 2.4 hours longer, whereas TAR decreased by 1.9 hours, with a significant decrease in mean HbA1c by 0.3% (3 mmol/mol) vs DSME alone [11]. These results clearly demonstrated that isCGM can improve glycemic outcomes. The present post hoc analysis demonstrated that age did not have an adverse impact on the benefits of isCGM in combination with DSME in a population using non-insulin agents to manage T2D. No significant differences were observed in TIR, TAR, or percentage of time spent in tight glycemic control between participants aged <65 and ≥65 years. In addition, no increases in diabetes distress were reported among older adults in our study. These findings suggest that older adults and their younger counterparts derive similar benefits from isCGM and DSME interventions. Further, these results challenge reported misconceptions that older individuals may not benefit from advanced technology due to factors such as dexterity or hearing limitations, cognitive impairment, or unfamiliarity with new diabetes devices such as CGMs [18].

Prevention of hypoglycemia is of particular importance when managing diabetes in older adults [2, 15]. Individuals on sulfonylureas are at an increased risk for hypoglycemic events due to the side effect profile of this medication. However, we found no significant increase in the frequency of hypoglycemic events in individuals aged ≥65 years, despite a higher proportion of participants in this age group using sulfonylureas (64.3%) than those <65 years (52.5%). Despite observing increases in percentage time spent in tight glycemic control, we did not find any significant difference in rates of hypoglycemia. These findings highlight the significance of combining isCGM and DSME as a way to achieve tighter glycemic control—as demonstrated in the main IMMEDIATE trial [11]—without exacerbating the risk of hypoglycemia in older adults. Our research parallels findings from the Wireless Innovation for Seniors with Diabetes Mellitus (WISDM) trial showing that older adults benefit from CGM technology. The WISDM authors reported that among adults aged ≥60 years with type 1 diabetes, CGM compared with standard blood glucose monitoring resulted in a significant improvement in hypoglycemia over 6 months [19]. Such findings may help encourage CGM uptake among older individuals. Although initiation of CGM devices has increased >10-fold among persons aged ≥65 years in the past decade, persons older than 75 years (and particularly those aged ≥85 years), along with racial and ethnic minorities and individuals from lower socioeconomic strata are less likely to be prescribed CGM than their counterparts [10]. Studies demonstrating the benefits of CGM may help counter these trends.

The GMSS score significantly improved with isCGM use in the younger age group, whereas older adults using isCGM did not report a significant increase in GMSS treatment satisfaction. Nevertheless, use of isCGM did not significantly increase diabetes distress as measured with the DDS in either age group, suggesting that isCGM use did not negatively impact older adults’ quality of life. The perceived usefulness of the CGM technology may have been tempered by age-related cognitive difficulties in interpreting glucose data and adjusting behavior accordingly or by physical limitations in manual dexterity or vision, which may have caused increased difficulty with reading screens or navigating the technology. The WISDM authors speculated that this may be due to low baseline scores reflecting fear of hypoglycemia and diabetes distress. Further investigation of older adults’ perceptions of the ease of use of isCGM would be of interest. For example, a qualitative study with interviews or focus groups might explore why older adults treated with isCGM + DSME may not have experienced significantly increased treatment satisfaction compared with those receiving DSME alone.

The limitations of the IMMEDIATE trial included its open-label design, lack of adjustment for multiple comparisons, and limited generalizability to individuals with better glycemic control or those managing their diabetes through lifestyle interventions alone. Additionally, factors such as blood glucose monitoring frequency and detailed assessments of diet and exercise were not fully explored. Since our post hoc analysis was based on data from the IMMEDIATE trial, these limitations also apply to our findings. In addition, the small sample size of the ≥65 years’ age group in our post hoc analysis limits the ability to detect significant age group by treatment group interactions.

## Conclusions

Our findings provide additional support for the use of isCGM and targeted DSME across a wide age spectrum of patients with T2D, including individuals aged <65 and ≥65 years of age.

## Data Availability

The data underlying this article will be shared on reasonable request to the corresponding author.

## Clinical Trial Information

Clinical trial registration number: NCT04562714.
